# Comparison of Distal Hypospadias Repair in Circumcised Patients and Uncircumcised Patients

**DOI:** 10.1155/2013/957581

**Published:** 2013-02-26

**Authors:** Fahimeh Kazemi Rashed, Rasool Gholizade

**Affiliations:** Urology Department, Imam Reza Hospital, Tabriz University of Medical Sciences, Tabriz 51666, Iran

## Abstract

Hypospadias is the most common anomaly in the male genital tract with an incidence of 0.8–8.2 per 1000 live male births. Routinely, hypospadias cases are repaired after one year of age, and it is recommended that a child with hypospadias not to be circumcised until hypospadias repair is completed. This study was conducted to determine whether or not circumcision prior to hypospadias repair increases the risk of complications. 30 circumcised patients and 30 persons without a history of circumcision and hypospadias were enrolled in this study and underwent surgery for hypospadias repair. The results of surgery compared between two groups. In the uncircumcised group, the mean durations of surgery and hospitalization were 55.61 ± 58.11 min and 3.17 ± 1.79 days. In circumcised group, the mean duration of surgery and hospitalization were 66.17 ± 33.65 minutes and 3.7 ± 1.62 days. There was no significant difference between these criteria and other complications of the two groups. This study shows that postoperative complications in circumcised distal hypospadias patients do not increase. Cosmetic and functional results were excellent. If there are failures in therapy and in case of postoperative complications, it is better to notice other factors such as infection.

## 1. Introduction

Hypospadias is the most common anomaly in male genital tract with approximately 0.8–8.2 per 1000 male newborns being affected [1]. Urethral hole in this anomaly is in ventral and proximal situation. This anomaly result from a failure of male urogenital folds to fuse in various regions. Hypospadias may exist alone, but usually is associated with chordee [2, 3]. Routinely, hypospadias cases are repaired after one year of age. And the traditional emphasis is that circumcision in hypospadiac males are postponed until the completion Hypospadias repair. This study compares the results of distal hypospadias repairs in circumcised patients with the results of uncircumcised patients to answer the question whether or not hypospadias repair complications increase in patients with hypospadias and a history of unknowingly or unwillingly circumcision prior to hypospadias repair.

## 2. Materials and Methods

Between September 2007–September 2010, 30 children with distal hypospadias and a history of circumcision and 30 uncircumcised children underwent TIP tubularized incised plate urethroplasty and used dartos flap as a second layer. The site of meatus is in distal of penis in all patients without chordee with complete or incomplete grove of glanse. All reconstructions were performed under general anesthesia, and the absence of chordee was confirmed by an artificial erection. The hypospadias was repaired by TIP urethroplasty method. Incised urethral plate was tubularized over a 10-F stent with continuous 6–0 vicryl sutures, and neourethra was covered with vascularized dartus flap harvested from dorsal penile skin in uncircumcised patients and from ventral penile skin in circumcised cases. Circumcision was performed in uncircumcised group, and the glandular wings and circumcision incision were repaired, and 10-F stent changed to a 8 F silicone stent that provided urine drainage for 2–4 days. All information about the age of circumcision, age of surgical repair, duration of surgery, hospitalization, and complications such that stenosis, fistulas, and penis appearance in six months after surgery in two groups were noted and compared. *t*-test was used to evaluate the quantitative data of the samples, and *U* Mann-Whitney test was used for qualitative data. Statistical analysis was down according to SPSS version 16. Statistically significant level of *P* is ≤0.05. This study been had approved by the Ethical Committee of the University.

## 3. Results and Discussion

60 patients were operated by one surgeon and were followed at least 6 months. In circumcised patients, mean age was 4.18 ± 2.49 years. Circumcisions were performed in the neonatal period except one that had been done in seven years old. Duration of surgery in this group was mean 65.33 ± 17.66 minutes. The mean hospitalization in this group was 3.8 ± 1.62 days (Table 1). In uncircumcised patients, mean age was 2.89 ± 2.56 years. The average duration of surgery was 61.55 ± 11.58 minutes. The average duration of hospitalization was 3.17 ± 1.79 days.

Severity of postoperative irritative symptoms was classified as mild spasms, moderate spasm (irritative symptoms and occasionally urinary leak around catheter), and severe spasms (irritative symptoms and persistent leak resulting in severe restlessness of child).

Spasm was zero in 2, mild in 23, and moderate in 5 circumcised patients.

**Figure 1:**
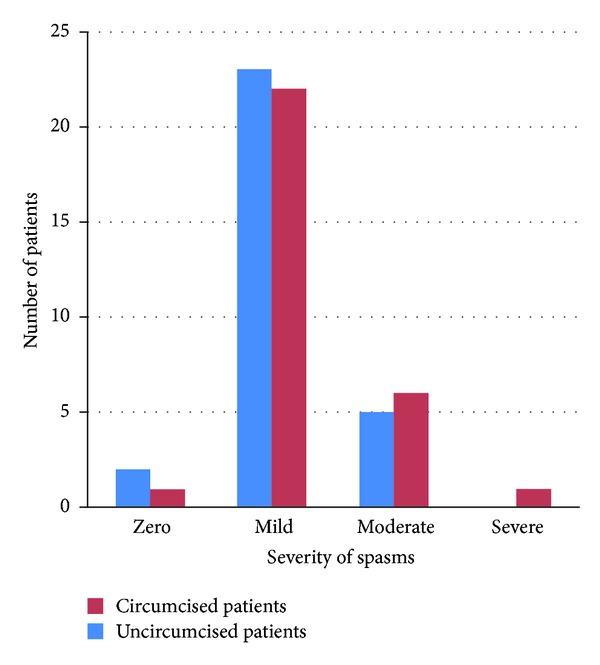
severity of bladder spasms between 2 groups.

There was zero spasm in one pakeat, mild in 22 patients, moderate in 6 patients, and severe in 1 patient (Figure 1).

**Figure 2:**
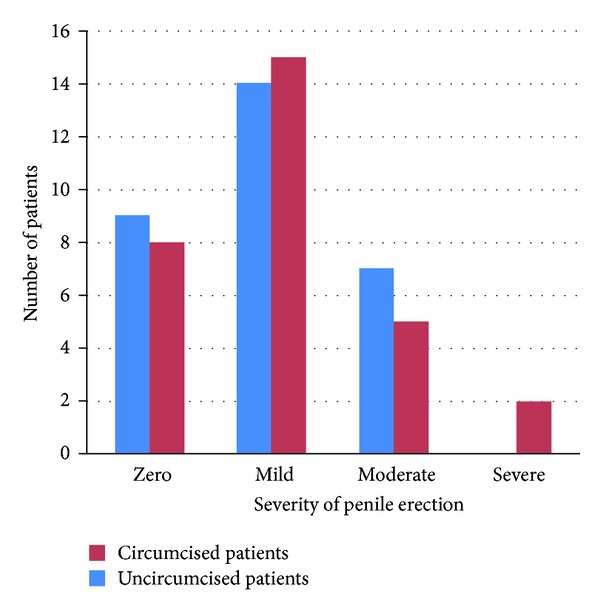
severity of penile erection between 2 groups.

Erection after surgery may increase also the rate and severity of complications, and it was classified into mild, moderate and severe. In the circumcised group, erection was zero in 9, mild in 14, and moderate in 7 patients. In uncircumcised patients erection composed zero in eight patients, mild in 15 cases, moderate in five cases, and severe in 2 patients (Figure 2).

We showed that comparison of bladder spasms (*P* = 0.97) and erection (*P* = 0.74) of two groups of patients are not significantly different. In control of complications after 6 months only, one case required meatoplasty for meatus stenosis in circumcised patients, and one case needed repair for urinary fistula in uncircumcised patients so the amount of surgical intervention to control of side effects in both groups was equal. The second surgery was performed 6 months after first surgery.

In this study, 60 patients with hypospadias anomaly in the two groups with and without a history of circumcision were studied. The aim of this study was to determine whether or not circumcision prior to hypospadias repair increases the risk of complications or all the complications in circumcised hypospadiac patients are related to previous circumcision.

In 1975, the American Academy of Pediatrics policy statement on circumcision described hypospadias as an absolute contraindication to the procedure. In 1999, the Academy reevaluated its 1975 and 1989 policies released new statement to perform circumcision after or synchronously with hypospadias repair [4]. Now, it is well accepted that infants with visible penile anomalies like hypospadias should wait for circumcision until the reconstructive surgery for hypospadias. This procedure is available with acceptable clinical outcomes within the first year of life on an outpatient basis because of improvement in anesthetic techniques, instrumentation, sutures dressing materials, and antibiotics. In spite of this protocol there are some cases of hypospadias that have been circumcised or diagnosed after circumcision, and it is needed to have a reasonable concept about the success rate and complication. 

In an article, Dr. Pieretti et al., evaluated the circumcised hypospadias patients. His group retrospectively reviewed 48 consecutive boys who had been diagnosed with hypospadias and had also been previously circumcised. In all the cases, the hypospadias was corrected with either urethral plate tubularization or MAGPI type of procedure. They did not utilize any skin flaps. All the patients were followed for at least eight months. Their findings were that prior circumcision did not negatively affect the results of their hypospadias repair. They felt that the use of a Snodgrass type of procedure has virtually eliminated the need for skin flaps for these repairs [5]. Despite the ways of performing these studies the results of both are the same.

Mousavi has studied 17 children with the average of 6.4 years who referred for surgery of hypospadias. Some patients were circumcised previously. Patients underwent TIP urethroplasty, and results and complications were studied. Complication was observed in four cases. 2 had stenosis of meatus, 1 had fistula and one had wound dehiscence. Surgery for such a case was necessary, and the remaining patients were treated with dilation. No complications related to prior circumcision in patients were observed in this study [6].

Snodgrass has studied retrospectively patients who had hypospadias and an intact prepuce; he compared patients who circumcised before diagnosis of defects of urethra and who had not been circumcised. Results showed that prior circumcision had not been complicate subsequent hypospadias repair in males whose urethral anomaly was concealed by an intact prepuce [7]. Our cases thoroughly consider Snodgrass results.

Leclair and colleagues in their paper report Foreskin reconstruction have decreased complication rates did not hypospadias distal repaired and that is recommended for all surgeons [8].

Djakovic et al. in an article has written as hypospadias explains various Hypospadias and comprehensive treatment for each patient. Although Many challenges exist in treating this disease. TIP Snodgrass method is widely used (in the distal Hypospadias repair). It is noticeable that have development of surgical methods and tools of modern surgery are considerable in the reduction of complications of surgery [9].

A very important point is that the used surgical techniques and skills of the surgeon can affect the healing results, but in our study all patients, surgeon was the same. Overall, it seems that our study's hypotheses regarding circumcision does not increase distal Hypospadias complication rate is correct and consistent with the fact that is international studies.

## 4. Conclusions

Reliable sources and references have emphasized that circumcision should be avoided in boys with hypospadias, but it seems that circumcision doesn't complicate repair of distal Hypospadias. It is better in complication situation the other causes also evaluated.

## Figures and Tables

**Table 1:** Mean and standard deviation of variables in the study groups.

	Circumcised group	Uncircumcised group	*P*
Age (year)	4.18 ± 2.49	2.89 ± 2.56	*P* = 0.09
Duration of surgery (minutes)	65.33 ± 17.66	61.55 ± 11.58	*P* = 0.33
Duration of hospitalization (days)	3.8 ± 1.62	3.17 ± 1.79	*P* = 0.16
Need to second surgery	1	1	—
